# Proton Acceleration with Relativistic Electromagnetic Shock

**DOI:** 10.1002/advs.202503538

**Published:** 2025-06-05

**Authors:** Ting Xiao, Xiaomei Zhang, Fanqiu Kong, Xiaolong Zheng, Zheng Gong, Baifei Shen

**Affiliations:** ^1^ Department of Physics Shanghai Normal University Shanghai 200234 China; ^2^ Institute of Theoretical Physics Chinese Academy of Sciences Beijing 100190 China

**Keywords:** intense laser pulse, proton acceleration, relativistic electromagnetic shock

## Abstract

Understanding the mechanisms behind the extreme energies of cosmic rays is crucial for unraveling fundamental physical processes in astrophysical environments. This study proposes a novel mechanism for accelerating cosmic‐ray protons. By examining a high‐velocity collision between an astrophysical object and static magnetic fields, the generation of an intense transverse electric field capable of trapping and accelerating protons are find to relativistic energies. Through Hamiltonian analysis, a scaling law that correlates the proton energy is derived to the minimum longitudinal thickness of the relativistic electromagnetic shock required for acceleration. One‐dimensional (1D) Particle‐In‐Cell (PIC) simulations show that an electromagnetic shock driver with a given intensity can accelerate protons from 4.7 MeV to 13 GeV, driven by the transverse electric field induce by the compressed static magnetic field. These results suggest that this mechanism can be experimentally realized in magnetized laser‐plasma systems, offering a novel approach for studying astrophysical phenomena in controlled laboratory experiments.

## Graphical abstract

**New Ion Acceleration Mechanism!** A novel cosmic‐ray proton acceleration mechanism is proposed, where relativistic electromagnetic shocks accelerate protons transversely. Hamiltonian analysis yields a scaling law linking the proton energy to the minimum longitudinal thickness of the shock. One‐dimensional Particle‐In‐Cell simulations demonstrate that a shock driver of fixed intensity can accelerate protons from 4.7 MeV to 13 GeV.

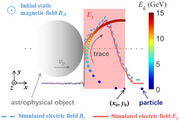

## 1 Introduction

The study of high‐energy cosmic rays is of significant interest to scientists, as it offers unique insights into the universe's most extreme and energetic phenomena. The energies carried by high‐energy cosmic rays far exceed those achievable in terrestrial laboratories, enabling the exploration of particle physics in a regime that may reveal physics beyond the Standard Model.^[^ ^1^, ^2^, ^3^, ^4^ ^]^ Recent advancements in observation techniques have greatly enhanced astrophysics and space science,^[^ ^5^, ^6^, ^7^, ^8^ ^]^ providing critical information about the composition, structure, and evolution of astrophysical systems.^[^ ^9^, ^10^, ^11^, ^12^ ^]^

Numerous theoretical models have been developed to explain the origin of high‐energy cosmic rays in the universe.^[^ ^13^, ^14^, ^15^, ^16^, ^17^, ^18^ ^]^ In extreme environments such as supernova remnants (SNRs), active galactic nuclei (AGNs), black holes, and pulsars, strong magnetic fields and shock waves are believed to play pivotal roles in particle acceleration.^[^ ^19^, ^20^, ^21^, ^22^, ^23^, ^24^, ^25^, ^26^ ^]^ Observations of SNRs, for example, indicate that charged particles can be accelerated to energies exceeding 100 TeV, consistent with relativistic shock models.^[^ ^27^ ^]^ The first‐order Fermi acceleration mechanism, driven by interactions between Alfven waves and converging plasma flows, is widely considered a primary explanation for particle acceleration in SNR shocks, both within the Milky Way and in extragalactic sources.^[^ ^28^ ^]^ On larger scales, such as in galaxy clusters and AGNs, the magnetic field is thought to be the primary driver of high‐energy particle acceleration.^[^ ^29^ ^]^ The cyclotron auto‐resonance acceleration mechanism has been proposed to explain proton acceleration when the magnetic and radiant fields are strictly parallel in binary systems merging or in supernova explosions.^[^ ^30^ ^]^ The compressible turbulence generated by galaxy cluster mergers can re‐accelerate electrons to high energies, triggering observable non‐thermal radiation.^[^ ^31^ ^]^ In interstellar matters, quasi‐perpendicular shock waves are employed to explain proton acceleration.^[^ ^32^, ^33^, ^34^ ^]^ Moreover, the diffusive shock acceleration mechanism in galaxy clusters operates on larger spatial scales and produces higher particle energies than SNRs.^[^ ^35^ ^]^

Previous studies demonstrate that the synergy between magnetic fields and shock waves is the key factor leading to particle acceleration in astrophysical environments and is essential for understanding the origin and energy distribution of cosmic rays.^[^ ^36^, ^37^, ^38^, ^39^ ^]^ Understanding how protons attain such high energies provides insights into the fundamental physical processes occurring in these extreme environments, facilitating the exploration of the fundamental forces of nature, particle interactions, and the behavior of matter under extreme conditions.^[^ ^40^, ^41^, ^42^, ^43^ ^]^ The interaction of high‐energy protons with interstellar matter can provide valuable insights into the structure of galaxies, cosmic magnetic fields, and cosmic dynamics.^[^ ^44^, ^45^, ^46^, ^47^ ^]^ Therefore, understanding proton acceleration mechanisms is crucial for exploring the origins of cosmic rays and unraveling the mysteries of high‐energy phenomena in the universe.^[^ ^48^, ^49^, ^50^, ^51^ ^]^

In this article, we propose a new mechanism for proton acceleration driven by high‐velocity collisions between astrophysical objects and static magnetic fields. Due to the conservation of magnetic flux,^[^ ^52^, ^53^ ^]^ the magnetic field is compressed by the induced plasma current from the object. In astrophysical environments, the scenario of objects with transverse sizes of 10^5^ to 10^20^ meters and speeds exceeding 0.9*c* compressing magnetic fields with strength from 10^−10^ to 10^−5^ T could take place in gamma‐ray bursts afterglow shocks^[^ ^54^, ^55^, ^56^, ^57^ ^]^ or relativistic jets exploded from AGNs.^[^ ^26^, ^58^, ^59^, ^60^ ^]^ Here, *c* is the speed of light in the vacuum. The strong transverse electric field is induced as the strong magnetic field can move at a velocity close to the speed of light. This electric field, in conjunction with the magnetic field, can trap and accelerate the injected protons to relativistic energies. Using Hamiltonian analysis,^[^ ^61^, ^62^ ^]^ we derive a scaling law that connects the maximum energy *E*_max_ of the accelerated protons to the relativistic factors γ_*p*_ associated with the drift motion of the astrophysical object and the initial relativistic factor γ_0_ of the accelerated protons. Additionally, we derive the criterion for the minimum longitudinal thickness *d* of the electromagnetic shock required to achieve proton trapping.

This mechanism provides a method for achieving high‐energy proton beams in laboratory laser‐plasma experiments. This mechanism has excellent scalability and promises to accelerate protons up to multi‐GeV. Given that the propagation direction of the driving laser pulse is perpendicular to the proton acceleration direction, this mechanism is ideal for coupling with multiple stages to achieve cascade proton acceleration, where the long‐distance transverse drifting motion could be realized by employing a transverse flying focus configuration.^[^ ^63^ ^]^

## 2 Results and Discussion

### 2.1 Theoretical Model

**Figure 1:**
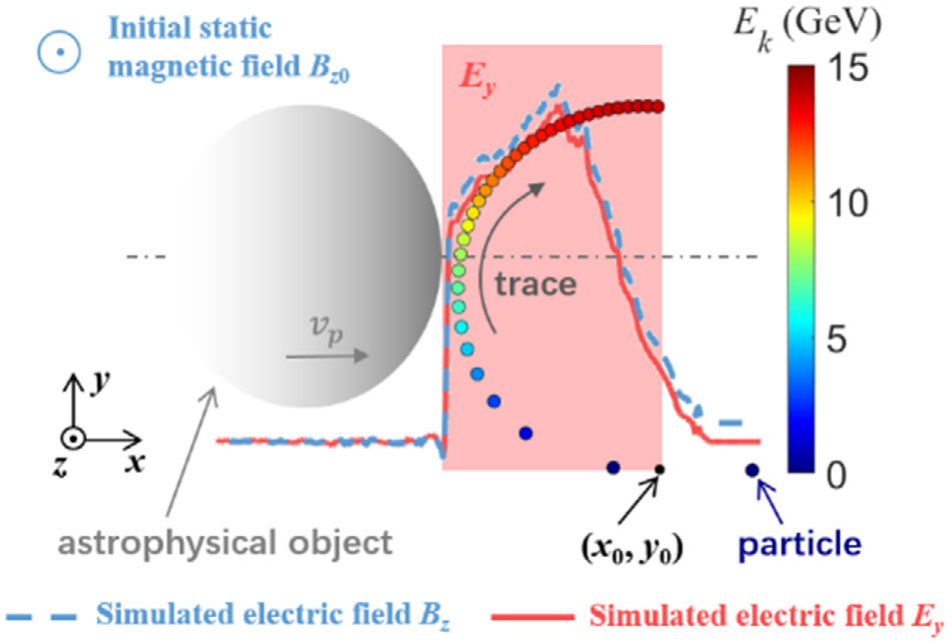
Schematic of Proton Acceleration. The astrophysical object colliding with magnetic fields compresses the field due to flux conservation, generating a transverse electric field that accelerates protons transversely. The colored circles show the trajectory of the accelerated proton.

When an astrophysical object moves with velocity *v_p_* (normalized to the speed of light *c*) along the *x*‐direction and collides with a static magnetic field *B*_*z*0_ in the perpendicular direction along *z* axis, the magnetic field is compressed due to the conservation of magnetic flux.^[^ ^52^, ^53^ ^]^ If the velocity of the object is relativistic, the relationship between the compressed magnetic field *B_z_* and the static magnetic field *B*_*z*0_ is given by $B_{z}=2\gamma_{p}^{2}B_{z0},$ ^[^ ^64^ ^]^ where the relativistic factor is $\gamma_{p}=1/\sqrt{1-v_{p}^{2}}\;$. The fast variation in the magnetic field generates a strong transverse electric field *E_y_*, which is given by  *E_y_* =  *B_z_v_p_*.^[^ ^64^ ^]^ Protons can be accelerated (or decelerated) transversely by this electric field *E_y_* (in pink‐shadowed region, transverse electric field assumed to be uniform in the *x*‐direction), as shown in **Figure** **1**.

We present a theoretical model of proton motion in the electromagnetic field. The coordinate in the co‐moving frame of the astrophysical object can be written as ξ  =  *x* − *v_p_t*. During the motion, the proton is transversely accelerated in the *y*‐direction by the electric field, while the magnetic field deflects it in the longitudinal direction. We have *A_x_* =   − *yB_z_* and φ  =   − *yE_y_*, where  *E_y_* =  *B_z_v_p_*, with *v_p_* being the velocity of the astrophysical object (normalized to the speed of light *c*). Since the magnetic field only alters the direction of the proton's motion, the longitudinal canonical momentum is conserved, *P_cx_* = *P*_*cx*0_ , where *P_cx_*_0_ is the proton's initial longitudinal canonical momentum. Assuming the proton's initial transverse position is *y*_0_ =  0 (as marked in Figure 1), *P_cx_* = *P*_*cx*0_  = *p*_*x*0_ , where *p*_*x*0_ is the proton's initial momentum in the *x*‐direction. The proton's momentum in the *x*‐direction is *p_x_* = *p*_*x*0_  + *a_x_y*β. β  = *m_e_* / *m_i_* =  1/1836 and *m_e_* (*m_i_*) is electron (proton) mass, *β* refers to the ratio of electron mass to the proton mass. For convenience, we normalize the electric field *E_y_* as *a_y_* = *E_y_* *e*/(*m_e_* ω_0_ *c*), with *e* being the proton charge, and the magnetic field *B_z_* as *a_x_* = *B_z_* *e*/(*m_e_* ω_0_), where ω_0_ =  2π*c*/λ_0_ is the angular frequency, with λ_0_ =  0.8 µm being the laser wavelength in our simulation. The coordinate *y* is normalized as *k*_0_ = λ_0_ /(2π).

By using Hamiltonian analysis, the derivation of the proton's momentum in the *y*‐direction as

(1) $$p_{y}=\sqrt{{\left(\gamma_{0}+a_{y}y\beta \right)}^{2}-1-p_{x}^{2}}\;$$

which is the function of transverse acceleration distance *y*. γ_0_ refers to the initial Lorentz factor of the proton. The momentum of the proton is correspondingly expressed as $p=\sqrt{{\left(\gamma_{0}+a_{y}y\beta \right)}^{2}-1}\;$. We can derive the normalized quantities from $a_{x}=2\gamma_{p}^{2}a_{x0}$ and *a_y_* = *a_x_* *v_p_*. Here, *a*_*x*0_ is the initial magnetic field normalized by *a*_*x*0_ = *B*_*z*0_ *e*/(*m_e_* ω_0_), where *B*_*z*0_ is the initial magnetic field.

**Figure 2:**
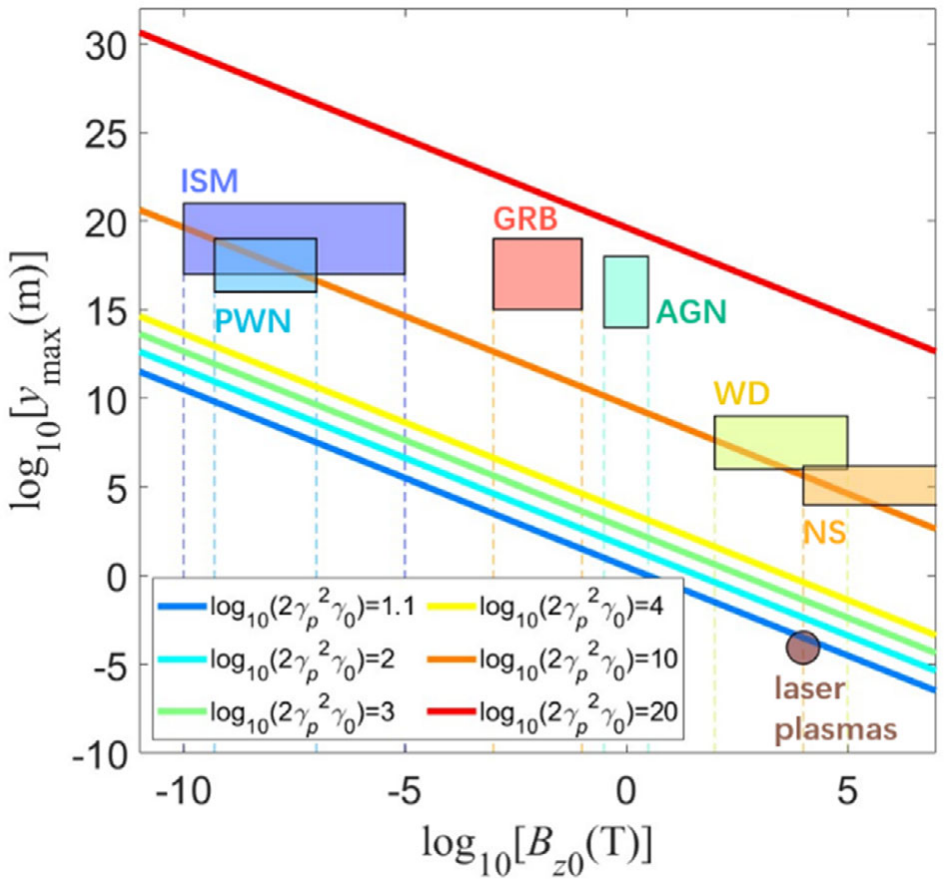
The relationship between the proton's maximum acceleration distance and the initial magnetic field strength with the maximum energy. The lines show the relationship between the proton's maximum acceleration distance *y*_max_ and the magnetic field strength *B*_*z*0_, with the velocity of the astrophysical object *v*_*p*_ = 0.925. The color area shows the representative magnetic field strengths and distance ranges for several astrophysical objects such as interstellar matter (ISM), pulsar wind nebulae (PWN), Gamma‐ray burst (GRB), active galactic nucleus (AGN), white dwarf stars (WD), and neutron stars (NS). The scale size of the magnetic fields of these objects is beyond the maximum acceleration distance *y*_max_.

As the proton can be deflected with the strong magnetic field induced by conservation of magnetic flux, the proton's transverse acceleration distance changed by both Lorentz force and strong transverse electric field. When *p_y_* =  0 in Equation (1), the acceleration process terminates, and the proton reaches the position (*x*_2_, *y*_2_). Given that the proton's initial position is *y*_0_ =  0, the maximum acceleration distance *y*_max_ can be determined. By normalizing the maximum acceleration distance *y*_max_ in meters (m) and formulating the magnetic field *B*_*z*0_ in Tesla (T), the relationship between the maximum acceleration distance and the magnetic field strength is given by

(2) $$y_{\max}\;\left(m\right)=\frac{v_{p}\gamma_{0}-p_{x0}+\sqrt{{\left(v_{p}\gamma_{0}-p_{x0}\right)}^{2}+p_{y0}^{2}/\gamma_{p}^{2}}}{B_{z0}\left(T\right)}\;\frac{m_{e}c}{2\beta e}$$

as shown in **Figure** **2**. If the velocity of the astrophysical object *v_p_*/*c* is close to unit, the maximum acceleration distance is approximated as $y_{\max}\approx \frac{\gamma_{0}-p_{x0}}{a_{x0}\beta }$. From this, we can obtain the energy at point 2, which represents the maximum energy *E*_max_ reached after acceleration:

(3) $$E_{\max}\approx \gamma_{0}+2\gamma_{p}^{2}\left(\gamma_{0}-p_{x0}\right)$$

In the scenario that proton's initial proton momentum is extremely large like astrophysical object, we can describe the maximum energy mainly by $2\gamma_{p}^{2}\gamma_{0}$. The proton's maximum energy *E*_max_ is related to the initial longitudinal momentum *p*_*x*0_ not initial transverse momentum *p*_*y*0_ from Equation (3), the initial proton momentum in the *x*‐direction is denoted as *p*_*x*0_, while the initial proton momentum in the *y*‐direction *p*_*y*0_ are all zero. The initial energies of the proton are calculated as $\gamma_{0}=\sqrt{1+p_{x0}^{2}}\;$.

From Figure 2, The upper‐left portion represents the proton acceleration process in astrophysical environments. The lower‐right portion represents the same process in laser plasmas with higher magnetic field but extremely small distance range. The color area in Figure 2 shows the representative magnetic field strengths and distance ranges for several astrophysical objects as interstellar matter (ISM),^[^ ^65^ ^]^ pulsar wind nebulae (PWN),^[^ ^66^ ^]^ Gamma‐ray burst (GRB),^[^ ^54^ ^]^ active galactic nucleus (AGN),^[^ ^67^ ^]^ white dwarf stars (WD),^[^ ^68^ ^]^ and neutron star (NS).^[^ ^69^ ^]^ In our mechanism, the maximum proton energy can be approximated as $E_{\max}∼2\gamma_{p}^{2}\gamma_{0}$, where γ_*p*_ is the flying mirror's Lorentz factor and γ_0_ is the proton's initial Lorentz factor. Different strength ranges of the magnetic field correspond to different astrophysical environments, such as interstellar matter (ISM), pulsar wind nebulae (PWN), Gamma‐ray burst (GRB), active galactic nucleus (AGN), white dwarf stars (WD), and neutron star (NS). Most of them, in principle, could take place the proposed electromagnetic shock acceleration to generate protons with the energy of $2\gamma_{p}^{2}\gamma_{0}$.

The scale size of magnetic fields of these objects is beyond the maximum acceleration distance *y*_max_ required to attain the maximum energy *E*_max_ in our mechanism. In both scenarios, the energy gained through acceleration is nearly identical, indicating that the astrophysical acceleration mechanism can be simulated based on laboratory laser‐plasma experiments. It is difficult for us to simulate the real large spatial acceleration range to reach the maximum energy for the astrophysical environment. However, when the plasma kinetic energy density ε_*k*_ =  *n*(γ_*p*_ − 1)*m_e_c* ^2^ significantly exceeds the magnetic energy density ε_*B*_ = *B* ^2^ /(2µ_0_), the magnetic field can be fully compressed (as shown in Figure S1 in Supporting Information), giving rise to a transverse electric field. Here, *n* represents the plasma density, γ_*p*_ is the plasma Lorentz Factor, *m_e_* denotes the electron mass, *B* is the magnetic field strength, and µ_0_ stands for the vacuum magnetic permeability. With the magnetization ratio σ  =  ε_*B*_/ε_*k*_ ∼ 10^−2^ ≪ 1, this parameter serves as a scaling factor bridging astrophysical environments and laboratory laser‐plasma interactions for our acceleration mechanism. On the other hand, we can consider a short acceleration range to get a lower energy under the same magnetic field, as the dashed lines shown in Figure 2. The range of parameters that can be simulated by the laser plasma is around on the order of ≈µm and ≈10 kT, shown as the blue line ($\mathrm{lo}g_{10}\;\left(2\gamma_{p}^{2}\gamma_{0}\right)=1.1$) in Figure 2. The laser‐plasma interaction shows that the proton energy can approach up to 13 GeV. The acceleration distance corresponding to the magnetic field strengths of different astrophysical environments on the blue line is much smaller than that provided by the astrophysical environment, so we can simulate the proton acceleration process corresponding to the astrophysical environmental parameters on this line in the laboratory. For $\mathrm{lo}g_{10}\;\left(2\gamma_{p}^{2}\gamma_{0}\right)=2,3,4$ (shown as the light blue, green and yellow line in Figure 2, respectively), such acceleration lengths are expected to be achieved in laboratory by cascade acceleration in the future.

For protons to be trapped by the magnetic field and continuously accelerated by the transverse electric field, they must undergo longitudinal reflection. This implies that the electromagnetic shock has sufficient longitudinal thickness. Assume the proton enters the electric field at *t*_0_ =  0, *y*_0_ =  0, and *x*_0_ =  0, and is reflected at point 1. In the object's co‐moving frame, where ξ  =  *x* − *v_p_t*, when the proton's velocity in the *x*‐direction is *v*_*x*1_ = *v_p_* , the position at the reflection point 1 is $y_{1}=\frac{\gamma_{0}v_{p}-p_{x0}}{2a_{x0}\beta }\;$; From *p_x_* = *p*_*x*0_  + *a_x_y*β and Equation (1), the relationship between the proton's position ξ and *y* can be derived as $\xi =\frac{1}{2a_{x0}\beta \gamma_{p}^{2}}\;\left(\sqrt{p_{y0}^{2}}-\sqrt{-4\beta^{2}a_{x0}^{2}\gamma_{p}^{2}y^{2}+4\beta \left(\gamma_{0}v_{p}-p_{x0}\right)a_{x0}\gamma_{p}^{2}y+p_{y0}^{2}}\right)$. Then we can get the corresponding position at the reflection point as $\xi_{1}=\frac{1}{2a_{x0}\beta \gamma_{p}^{2}}\;\left(\sqrt{p_{y0}^{2}}-\sqrt{\gamma_{p}^{2}{\left(\gamma_{0}v_{p}-p_{x0}\right)}^{2}+p_{y0}^{2}}\right)$. Finally, by taking the negative of ξ_1_ and normalized in the micron (µm), we obtain the minimum longitudinal thickness *d* of the relativistic electromagnetic shock required for the proton to undergo reflection and continue the acceleration process:

(4) $$d\;\left(\mu m\right)=\frac{0.2}{\pi a_{x0}\beta \gamma_{p}^{2}}\;\left(\sqrt{\gamma_{p}^{2}{\left(v_{p}\gamma_{0}-p_{x0}\right)}^{2}+p_{y0}^{2}}-\sqrt{p_{y0}^{2}}\right)$$

**Figure 3:**
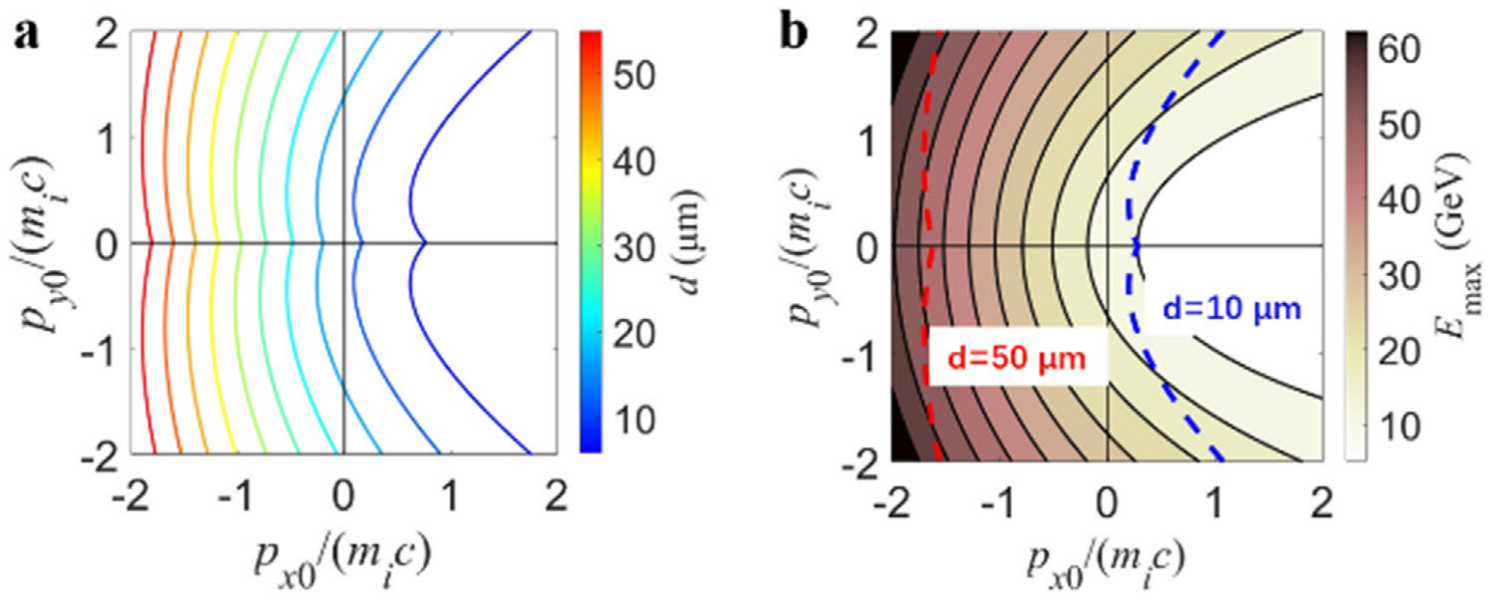
The trapped conditions and proton energy. a) The minimum longitudinal thickness d (shown by the color code) of the relativistic electromagnetic shock corresponding to different injected proton's initial momenta p_x0_ and *p*_*y*0_. b) The maximum proton energy *E*_max_ (shown by the color code) corresponding to different injected proton's initial momenta *p*_*x*0_ and *p*_*y*0_. The initial proton momentum with thickness of 10 µm (blue dashed line) and 50 µm (red dashed line) is marked on the plot, respectively.

From Figure 3b, a longitudinal thickness of approximately 50 µm for the electromagnetic shock to accelerate protons with this mechanism can be realized in laboratory experiments.

### 2.2 Simulation Validation

To understand this mechanism, we scale the parameters to a range suitable for laboratory simulation and perform the One‐dimensional (1D) Particle‐In‐Cell (PIC) simulations.

**Figure 4:**
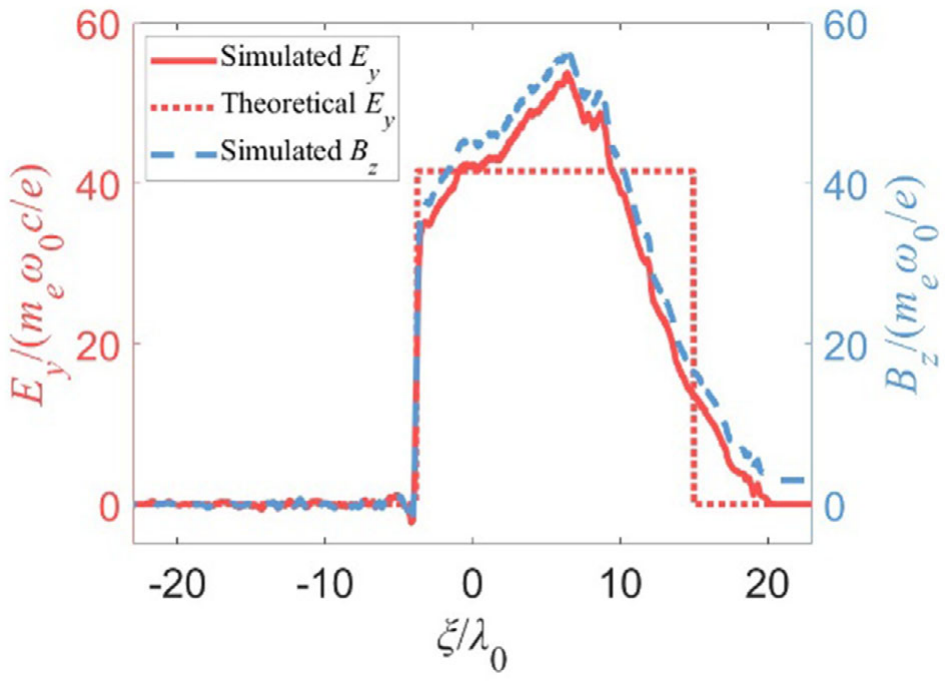
The relativistic electromagnetic shock induced by compressing a static magnetic field through interaction with a strong laser (*a*_0_ = 150) at 295T_0_.

In the relativistic flying mirror's^[^ ^64^ ^]^ co‐moving frame, where ξ  =  *x* − *v_p_t*, *v_p_* ≈ 0.925 is the speed of the relativistic flying mirror (obtained from the simulation results and normalized to the speed of light *c*). **Figure** **4** shows that when the strong laser interacts with the plasma, the static magnetic field *B*_z0_ =  40000 T ( *a*_*x*0_ = *B*_z0_ *e*/(*m_e_* ω_0_) ≈ 3) is compressed, generating a maximum transverse electric field *E_y_e*/(*m_e_* ω_0_ *c*) of nearly 60, as shown by the red solid line. The relationship between the electric field *E_y_* and the initial static magnetic field *B*_*z*0_ is expressed as $E_{y}=2\gamma_{p}^{2}B_{z0}c/v_{p}\;$. The red dotted line represents the theoretically achievable transverse electric field *E_y_*.

**Figure 5:**
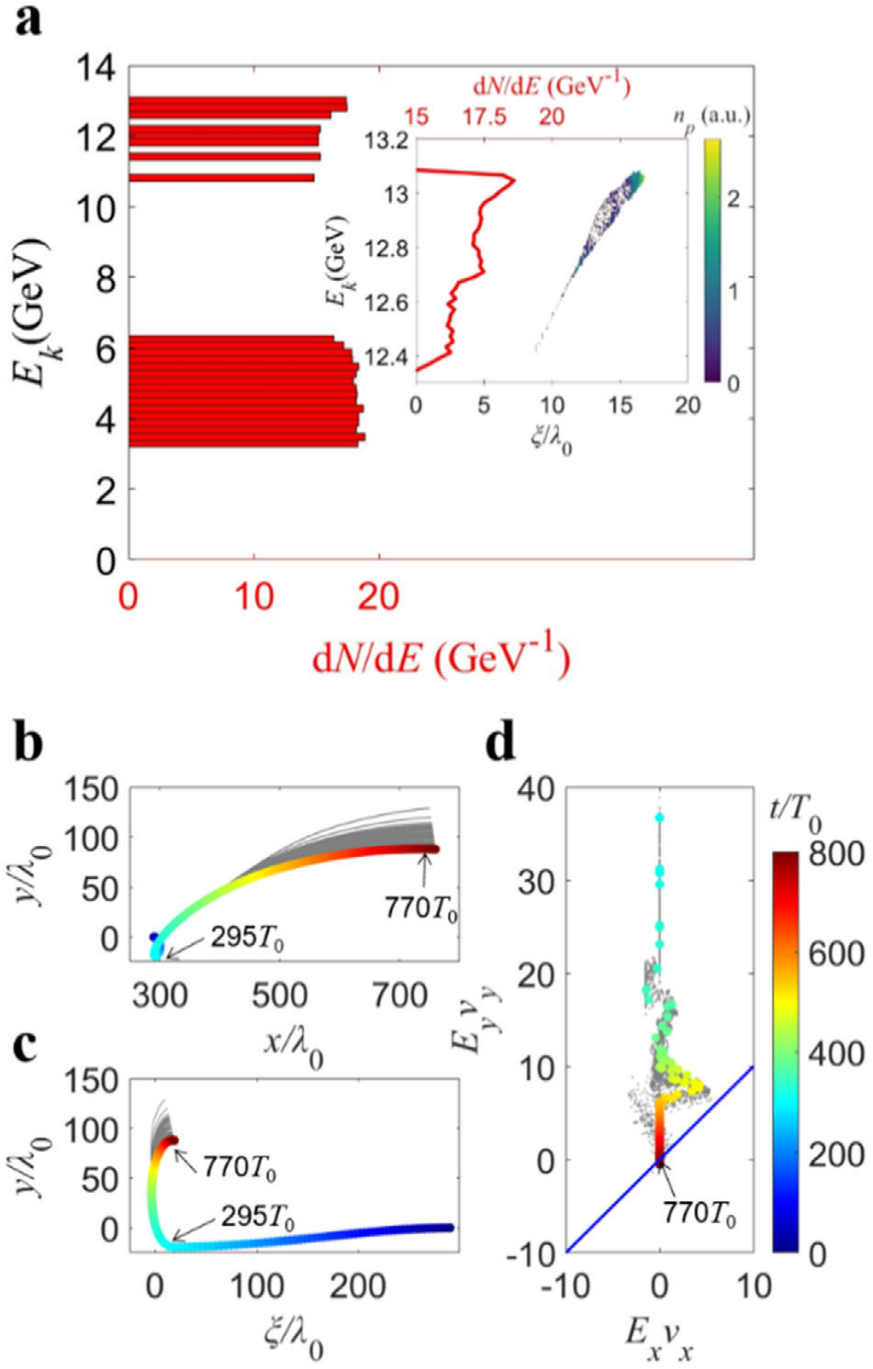
The acceleration result and process of the trapped protons. a) The energy distribution of protons at 770T_0_ after acceleration. The acceleration process of the trapped protons, with the color code representing the simulation time. b, c) The motion trajectories of the high‐energy protons in the *x* and *ξ* coordinate systems, separately. d) The energy source of the high‐energy protons.

The selected protons from the trapped high‐energy proton group in *x* (ξ) coordinate system are shown following the motion trajectories depicted in **Figure** **5**b,c. At the start of the simulation (0*T*_0_), a proton beam with an initial momentum *p*_*x*,in_ =  0.1 (normalized with respect to the proton's momentum *m_i_c*) and *p*_*y*,in_ =  0 is injected into the region 250λ_0_ < *x* < 300λ_0_, and its initial energy is approximately 4.7 MeV. After approximately 295*T*_0_ (about half cycle of the proton's motion in the 40000 T magnetic field), the proton beam is deflected by the initial static magnetic field *B*_*z*0_, altering its momentum to *p*_*x*0_ =   − 0.1, *p*_*y*0_ =  0. It then enters the relativistic electromagnetic shock, where it is accelerated by the transverse electric field. At *t*  =  770*T*_0_, the proton energy spectrum is shown in Figure 5a. The high‐energy portion of the spectrum results from the trapping and acceleration of protons by the intense transverse electric field *E_y_*. The inset shows the energy distribution of the high‐energy portion, where the red line represents the proton energy spectrum, and the color code represents the proton density distribution. This demonstrates that this process can accelerate protons from 4.7 MeV to 13 GeV. Preliminary 2D simulations (as shown in Figure S2 in Supporting Information) indicate that the flying mirror can last for 1 fs and accelerate protons.

The protons enter the transverse electric field *E_y_* at 295*T*_0_, traveling considerable distances in the transverse direction due to the acceleration (Figure 5b,c). The energy gained by the protons primarily originates from *E_y_* (Figure 5d), the transverse electric field generated by the compression of the static magnetic field due to the interaction between the strong laser and the plasma. After 770*T*_0_, the protons, influenced by the Lorentz force, acquire negative transverse momentum *p_y_*, leading to their deceleration and energy loss. As a result, the instantaneous power values fall below the blue line *y*  =  *x* in Figure 5d. The simulated acceleration length is *y*_max_ =  107λ_0_, where λ_0_ =  0.8 µm is the laser wavelength. The simulated proton energy is *E*_max_ =  13 GeV. Comparing the simulation result with the theoretically predicted value of *y*_max_ =  100λ_0_ and *E*_*k*2_ =  15.3 GeV, we find that the simulation results basically agree well with the predictions of the Hamiltonian analysis.

### 2.3 Discussion

The proposed electromagnetic shock acceleration requires an external magnetic field with a strength of 40000 T, which could be achievable in the near future. Laser‐plasma experiments have already realized kT‐level magnetic field,^[^ ^70^ ^]^ while there is some different opinion.^[^ ^71^ ^]^ The PIC simulations have generated magnetic fields as strong as megatesla.^[^ ^72^ ^]^ The relevant details of producing such a 40000 T magnetic field in the laboratory still need to be further studied. Considering the compression ratio for the magnetic field is much higher for the flying mirror with higher velocity, it is possible to realize the proposed electromagnetic shock acceleration based on the current magnetic field of kT‐level by applying a more intense laser pulse. Alternatively, the magnetic and electric fields can be further enhanced by using the double‐flying‐mirror scheme, where the magnetic field can be further compressed by another flying mirror in the opposite direction.

**Figure 6:**
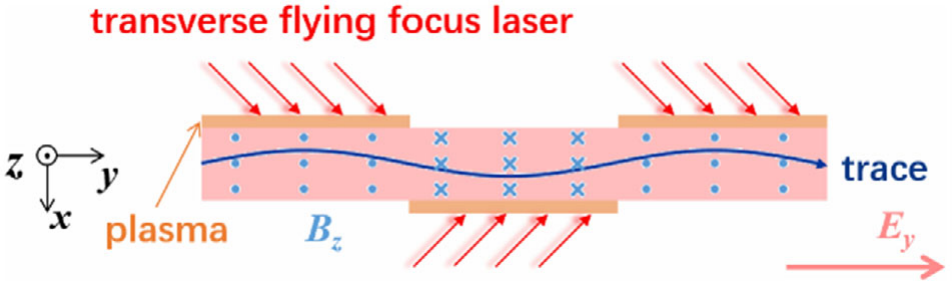
Schematic diagram of relativistic electromagnetic shock cascaded acceleration. The direction of the magnetic field needs to change according to the direction of motion of the proton so that the transverse electric field is always in the *y*‐direction.

To achieve longer acceleration distances and higher proton energies, multiple laser‐plasma modules can be used for cascaded acceleration, as shown in **Figure** **6**. In addition, using a transverse flying focus laser configuration^[^ ^63^ ^]^ can further optimize the acceleration effect. Electron acceleration can also be achieved with this method. It is worth emphasizing that the transverse flying focus technique can help reduce the required laser power to realize the extended transverse acceleration distance. Therefore, the proposed mechanism of electromagnetic shock proton acceleration is expected to be realized within the state‐of‐the‐art multi‐PW laser systems.

In addition, the acceleration mechanism of relativistic electromagnetic shock indicates that the maximum proton energy can approach to $E_{\max}∼2\gamma_{p}^{2}\gamma_{0}m_{i}c^{2}$. The observed proton energy of cosmic rays up to 10^18^ eV corresponds to $2\gamma_{p}^{2}\gamma_{0}∼10^{9}$. In Figure 2, the orange line of $\mathrm{lo}g_{10}\;\left(2\gamma_{p}^{2}\gamma_{0}\right)=10$ illustrates that the scaled parameters could be relevant to astrophysical scenarios of interstellar matter (ISM), pulsar wind nebulae (PWN), white dwarf stars (WD), neutron star (NS), and in the region of Gamma‐ray burst (GRB) and active galactic nucleus (AGN). For the magnetic field strength of the above astrophysical scenarios, the distance required for the proton's energy (the order of 10^18^ eV) is shorter than the scale length of these scenarios. Therefore, this mechanism could, in principle, take place in the above astrophysical scenarios considering the spatial scale and strength of their magnetic fields to realize the acceleration of ultra‐high‐energy cosmic‐ray particles with energies greater than 10^18^ eV.

## 3 Conclusion

In summary, this work shows a new cosmic‐ray protons acceleration mechanism in astrophysical environments, where the astrophysical object colliding with magnetic fields (plasma is not required) compresses the field due to flux conservation, generating a relativistic electromagnetic shock that accelerates protons transversely. Using Hamiltonian analysis, we analytically derived a scaling law that relates the energy of protons to the minimum longitudinal distance of the relativistic electromagnetic shock required for acceleration. 1D PIC simulations, using a laser pulse with an intensity of 9.6 × 10^22^ W/cm^2^ to interact with plasma and compress a 40000 T static magnetic field, demonstrate that the transverse electric field generated by this process can accelerate protons from 4.7 MeV to 13 GeV. Moreover, cascaded acceleration can increase the acceleration distance and reach higher proton energies. This acceleration mechanism not only provides a novel method for proton acceleration but also enables the simulation of high‐energy physical phenomena in astrophysical environments, thereby advancing laboratory astrophysics.

## 4 Experimental Section

### Hamiltonian Analysis

The Hamiltonian for proton motion in the electromagnetic field was derived as

(5) $$H=\sqrt{m_{i}^{2}c^{4}+{\left(P_{cx}-eA_{x}\left(y\right)\right)}^{2}c^{2}+p_{y}^{2}c^{2}}+e\varphi \left(y\right)$$

*m_i_* was the proton mass, *c* was the speed of light, *P_cx_* was the canonical momentum of the proton in the *x*‐direction, where *P_cx_* = *p_x_*  + *eA_x_*, with *e* being the proton charge and *A_x_* the vector potential in the *x*‐direction. *p_x_* and *p_y_* were the proton momenta in the *x*‐ and *y*‐directions, respectively, and φ was the scalar potential.

The Hamiltonian describing the proton's motion during the acceleration process was given by $\;H=\sqrt{m_{i}^{2}c^{4}+{\left(p_{x0}+eyB_{z}\right)}^{2}c^{2}+p_{y}^{2}c^{2}}\;-eyE_{y}$. After normalization, the expression becomes:

(6) $$H=\sqrt{1+{\left(p_{x0}+a_{x}y\beta \right)}^{2}+p_{y}^{2}}\;-a_{y}y\beta$$

*p*_*x*0_ was the initial *x*‐direction momentum of the proton, and *p_y_* was its *y*‐direction momentum, both normalized with respect to the proton's momentum *m_i_c*. The longitudinal vector potential *a_x_* was used to describe the transverse magnetic field *B_z_*, where *a_x_* = *B_z_* *e*/(*m_e_* ω_0_) was the normalized magnetic field strength. The proton was initially located at *y*_0_ =  0. The initial Hamiltonian *h*_0_ of the proton was derived as

(7) $$h_{0}=\sqrt{1+{p_{x0}}^{2}+p_{y0}^{2}}\;-a_{y}y_{0}\beta =\gamma_{0}\;$$

here *p*_*y*0_ was the initial *y*‐direction momentum of the proton. By using the conservation of the Hamiltonian during the acceleration process, Equation (6) and (7) were thus equivalent. This leads to the derivation of the proton's momentum in the *y*‐direction.

### PIC Simulation

The 1D PIC simulations using the completely relativistic code EPOCH was performed. A circularly polarized laser pulse was injected from the left boundary of the simulation window and propagates along the *x*‐axis. The pulse intensity was *I*_0_ ≈ 9.6 × 10^22^ W/cm^2^, corresponding to normalized amplitude of the laser *a*_0_ = *E*_0_ *e*/(*m_e_* ω_0_ *c*)  =  150, where *E*_0_ was the laser's electric field amplitude, ω_0_ =  2π*c*/λ_0_ was the angular frequency, and λ_0_ =  0.8 µm was the wavelength. The laser pulse has a trapezoidal temporal profile: 3*T*_0_ − 10*T*_0_ − 3*T*_0_, where *T*_0_ = λ_0_/*c*  was the laser cycle. A thin solid target was located at *x_m_* =  10λ_0_, and it was modeled as a hydrogen plasma with a thickness of *d_m_* =  240 nm and electron density *n_e_* =  200 *n_c_*, where $n_{c}\approx \varepsilon_{0}m_{e}\omega_{0}^{2}/e^{2}\approx 1.7\times 10^{21}\;cm^{-3}$ was the critical density, ε_0_ was the vacuum permittivity, *m_e_* was the electron mass, and *e* was the proton charge. The sharp plasma density boundary utilized here does not impact the effect of the proposed mechanism. In addition, the plasma here can be considered as collisionless ideal plasma, so magnetic diffusion can be negligible. A magnetic field with 40000 T along the *z*‐axis in the region *x* > 10λ_0_. The 1D simulations have a size of 300λ_0_, with a grid size of Δ*x*  = λ_0_/200 . 200 (50) macro‐particles were used per real particle for target (injected protons) in each grid, with open boundary conditions applied in the ± *x*‐direction. To speed up the simulation, the moving window technique was applied. It was worth emphasizing that in this 1D PIC simulation, the spatial grid was 1D, but electromagnetic fields (*E* and *B*) and particle velocities retain three components. This was crucial for capturing phenomena like magnetic field effects or particle acceleration in perpendicular directions. In addition, the normalized magnetic field *B* ≈ 4*m_e_* ω_0_/*e* and electron density *n_e_* =  200*n_c_*. For the scenario of laser‐plasma interaction, the typical laser frequency was ω_0_ =  2π*c*/λ_0_ where λ_0_ =  1 µm. For the situation of the ISM irradiated by astrophysical GRB outflows, if one chooses λ_0_ =  4 × 10^5^ m, the magnetic field strength can be estimated as *B* ≈ 4*m_e_* ω_0_/*e* ∼ 10^−7^ T and plasma density was approximately *n_e_* =  200*n_c_* −1.2 cm^−3^.

## Conflict of Interest

The authors declare no conflict of interest.

## Supporting information



Supporting Information

## Data Availability

All data needed to evaluate the conclusions of the paper are present in the paper.

## References

[1] A. W. Strong , I. V. Moskalenko , V. S. Ptuskin , Annu. Rev. Nucl. Part. Sci. 2007, 57, 285.

[2] K. Kotera , A. V. Olinto , Annu. Rev. Astron. Astrophys. 2011, 49, 119.

[3] R. A Batista , J. Biteau , M. Bustamante , K. Dolag , R. Engel , K. Fang , K.‐H. Kampert , D. Kostunin , M. Mostafa , K. Murase , F. Oikonomou , A. V. Olinto , M. I. Panasyuk , G. Sigl , A. M. Taylor , M. Unger , Front. Astron. Space Sci. 2019, 6, 35.

[4] M. Ruszkowski , C. Pfrommer , Astron. Astrophys. Rev. 2023, 31, 237. 10.1007/s00159-023-00149-2 PMC10730010 38115816

[5] J. L. Feng , Annu. Rev. Astron. Astrophys. 2010, 48, 495.

[6] F. G. Schröder , Prog. Part. Nucl. Phys. 2017, 93, 1.

[7] E. Petroff , J. W. T. Hessels , D. R. Lorimer , Astron. Astrophys. Rev. 2019, 27, 75. 10.1007/s00159-019-0116-6 PMC11557685 39544369

[8] M. Bailes , B. K. Berger , P. R. Brady , M. Branchesi , K. Danzmann , M. Evans , K. Holley‐Bockelmann , B. R. Iyer , T. Kajita , S. Katsanevas , M. Kramer , A. Lazzarini , L. Lehner , G. Losurdo , H. Lück , D. E. McClelland , M. A. McLaughlin , M. Punturo , S. Ransom , S. Raychaudhury , D. H. Reitze , F. Ricci , S. Rowan , Y. Saito , G. H. Sanders , B. S. Sathyaprakash , B. F. Schutz , A. Sesana , H. Shinkai , et al., Nat. Rev. Phys. 2021, 3, 344.

[9] R. Blandford , D. Eichler , Phys. Rep 1987, 154, 1.

[10] G. J. Fishman , C. A. Meegan , Annu. Rev. Astron. Astrophys. 1995, 33, 415.

[11] G. Dubus , Astron. Astrophys. Rev. 2013, 21, 71.

[12] R. Bühler , R. Blandford , Rep. Prog. Phys. 2014, 77, 066901. 24913306 10.1088/0034-4885/77/6/066901

[13] M. Hoshino , Astrophys. J. 2008, 672, 940.

[14] L. Sironi , A. Spitkovsky , Astrophys. J. 2011, 726, 75.

[15] P. Blasi , Astron. Astrophys. Rev. 2013, 21, 73.

[16] P. Kumar , B. Zhang , Phys. Rep 2015, 561, 1.

[17] A. Marcowith , A. Bret , A. Bykov , M. E. Dieckman , L. O. C. Drury , B. Lembège , M. Lemoine , G. Morlino , G. Murphy , G. Pelletier , I. Plotnikov , B. Reville , M. Riquelme , L. Sironi , A. S. Novo , Rep. Prog. Phys. 2016, 79, 046901. 27007555 10.1088/0034-4885/79/4/046901

[18] T. Amano , M. Hoshino , Astrophys. J. 2022, 927, 132.

[19] A P. Marscher , S G. Jorstad , F D. D'Arcangelo , P S. Smith , G. G Williams , V M. Larionov , H. Oh , A R. Olmstead , M F. Aller , H D. Aller , I M. McHardy , A. Lähteenmäki , M. Tornikoski , E. Valtaoja , V A. Hagen‐Thorn , E N. Kopatskaya , W K. Gear , G. Tosti , O. Kurtanidze , M. Nikolashvili , L. Sigua , H. R Miller , W T. Ryle , Nature 2008, 452, 966. 18432239 10.1038/nature06895

[20] B. Cerutti , D. A. Uzdensky , M. C. Begelman , Astrophys. J. 2012, 746, 148.

[21] J. Vink , Astron. Astrophys. Rev. 2012, 20, 49.

[22] A. Abramowski , F. Aharonian , F. Ait Benkhali , A. G. Akhperjanian , E. Angüner , G. Anton , S. Balenderan , A. Balzer , A. Barnacka , Y. Becherini , J. Becker Tjus , K. Bernlöhr , E. Birsin , E. Bissaldi , J. Biteau , M. Böttcher , C. Boisson , J. Bolmont , P. Bordas , J. Brucker , F. Brun , P. Brun , T. Bulik , S. Carrigan , S. Casanova , M. Cerruti , P. M. Chadwick , R. Chalme‐Calvet , R. C G Chaves , A. Cheesebrough , et al., Astron. Astrophys. 2014, 562, L4.

[23] J. Aleksic , S. Ansoldi , L. A. Antonelli , P. Antoranz , A. Babic , P. Bangale , J. A. Barrio , J. B González , W. Bednarek , E. Bernardini , B. Biasuzzi , A. Biland , O. Blanch , S. Bonnefoy , G. Bonnoli , F. Borracci , T. Bretz , E. Carmona , A. Carosi , P. Colin , E. Colombo , J. L. Contreras , J. Cortina , S. Covino , P. Da Vela , F. Dazzi , A. De Angelis , G. De Caneva , B. De Lotto , E. D. O. Wilhelmi , et al., Science 2014, 346, 1080. 25378461 10.1126/science.1256183

[24] M D. Johnson , V L. Fish , S S. Doeleman , D P. Marrone , R L. Plambeck , J F. C. Wardle , K. Akiyama , K. Asada , C. Beaudoin , L. Blackburn , R. Blundell , G C. Bower , C. Brinkerink , A E. Broderick , R. Cappallo , A A. Chael , G B. Crew , J. Dexter , M. Dexter , R. Freund , P. Friberg , R. Gold , M A. Gurwell , P T. P. Ho , M. Honma , M. Inoue , M. Kosowsky , T P. Krichbaum , J. Lamb , A. Loeb , et al., Science 2015, 350, 1242. 26785487 10.1126/science.aac7087

[25] J. J. Kroon , P. A. Becker , J. D. Finke , C. D. Dermer , Astrophys. J. 2016, 833, 157.

[26] R. Blandford , D. Meier , A. Readhead , Annu. Rev. Astron. Astrophys. 2019, 57, 467.

[27] F. A. Aharonian , A. G. Akhperjanian , K.‐M. Aye , A. R. Bazer‐Bachi , M. Beilicke , W. Benbow , D. Berge , P. Berghaus , K. Bernlöhr , O. Bolz , C. Boisson , C. Borgmeier , F. Breitling , A. M. Brown , J. Bussons Gordo , P. M. Chadwick , V. R. Chitnis , L.‐M. Chounet , R. Cornils , L. Costamante , B. Degrange , A. Djannati‐Ataï , L. O'C. Drury , T. Ergin , P. Espigat , F. Feinstein , P. Fleury , G. Fontaine , S. Funk , Y. A. Gallant , et al., Nature 2004, 432, 75. 15525982 10.1038/nature02960

[28] R. D. Blandford , J. P. Ostriker , Astrophys. J. 1978, 221, L29.

[29] L. Sironi , M. Petropoulou , D. Giannios , Mon. Not. R. Astron. Soc. 2015, 450, 183. 10.1093/mnras/stw749 27279781

[30] Y. I. Salamin , M. Wen , C. H. Keitel , Astrophys. J. 2021, 907, 24.

[31] G. Brunetti , A. Lazarian , Mon. Not. R. Astron. Soc. 2007, 378, 245.

[32] R. H. Burrows , G. P. Zank , G. M. Webb , L. F. Burlaga , N. F. Ness , Astrophys. J. 2010, 715, 1109.

[33] G. P. Zank , J. Heerikhuisen , N. V. Pogorelov , R. Burrows , D. McComas , Astrophys. J. 2010, 708, 1092.

[34] S. V. Chalov , Y. G. Malama , D. B. Alexashov , V. V. Izmodenov , Mon. Not. R. Astron. Soc. 2016, 455, 431.

[35] R. J. Van Weeren , H. J. A. Röttgering , M. Brüggen , M. Hoeft , Science 2010, 330, 347. 20929732 10.1126/science.1194293

[36] F. Fiuza , R. A. Fonseca , J. Tonge , W. B. Mori , L. O. Silva , Phys. Rev. Lett. 2012, 108, 235004. 23003965 10.1103/PhysRevLett.108.235004

[37] W. Yao , A. Fazzini , S. N. Chen , K. Burdonov , P. Antici , J. Béard , S. Bolaños , A. Ciardi , R. Diab , E. D. Filippov , S. Kisyov , V. Lelasseux , M. Miceli , Q. Moreno , V. Nastasa , S. Orlando , S. Pikuz , D. C. Popescu , G. Revet , X. Ribeyre , E. d'Humières , J. Fuchs , Nat. Phys. 2021, 17, 1177.

[38] Z. Gong , X. Shen , K. Z. Hatsagortsyan , C. H. Keitel , Phys. Rev. Lett. 2023, 131, 225101. 38101383 10.1103/PhysRevLett.131.225101

[39] G. Zhang , Z. Sheng , S. Weng , M. Chen , J. Zhang , Chin. Phys. B 2024, 33, 115115203.

[40] X. Zhang , B. Shen , X. Li , Z. Jin , F. Wang , M. Wen , Phys. Plasmas 2007, 14, 123108.

[41] R. R. Caldwell , M. Kamionkowski , Annu. Rev. Nucl. Part. Sci. 2009, 59, 397.

[42] F. Wagner , O. Deppert , C. Brabetz , P. Fiala , A. Kleinschmidt , P. Poth , V. A. Schanz , A. Tebartz , B. Zielbauer , M. Roth , T. Stöhlker , V. Bagnoud , Phys. Rev. Lett. 2016, 116, 205002. 27258872 10.1103/PhysRevLett.116.205002

[43] H. Zhang , B. F. Shen , W. P. Wang , S. H. Zhai , S. S. Li , X. M. Lu , J. F. Li , R. J. Xu , X. L. Wang , X. Y. Liang , Y. X. Leng , R. X. Li , Z. Z. Xu , Phys. Rev. Lett. 2017, 119, 164801. 29099228 10.1103/PhysRevLett.119.164801

[44] E. G. Zweibel , J. E. Everett , Astrophys. J. 2010, 709, 1412.

[45] T. M. Yoast‐Hull , J. S. Gallagher , E. G. Zweibel , Astrophys. J. 2014, 790, 86.

[46] R. J. Van Weeren , F. De Gasperin , H. Akamatsu , M. Brüggen , L. Feretti , H. Kang , A. Stroe , F. Zandanel , Space Sci. Rev. 2019, 215, 75.

[47] J. H. Hamer , K. C. Schlaufman , Astron. J. 2020, 160, 138.

[48] L. O. C. Drury , Astropart. Phys 2012, 39, 52.

[49] A. Macchi , M. Borghesi , M. Passoni , Rev. Mod. Phys. 2013, 85, 751.

[50] L. Sironi , A. Spitkovsky , Astrophys. J. 2014, 783, L21.

[51] T. Ebisuzaki , T. Tajima , Astropart. Phys 2021, 128, 102567.

[52] O. V. Gotchev , P. Y. Chang , J. P. Knauer , D. D. Meyerhofer , O. Polomarov , J. Frenje , C. K. Li , M. J. E. Manuel , R. D. Petrasso , J. R. Rygg , F. H. Séguin , R. Betti , Phys. Rev. Lett. 2009, 103, 215004. 20366046 10.1103/PhysRevLett.103.215004

[53] J. P. Knauer , O. V. Gotchev , P. Y. Chang , D. D. Meyerhofer , O. Polomarov , R. Betti , J. A. Frenje , C. K. Li , M. J. E. Manuel , R. D. Petrasso , J. R. Rygg , F. H. Séguin , Phys. Plasmas 2010, 17, 056318. 10.1103/PhysRevLett.103.215004 20366046

[54] M. V. Medvedev , A. Loeb , Astrophys. J. 1999, 526, 697.

[55] T. Piran , Rev. Mod. Phys. 2005, 76, 1143.

[56] B. Zhang , Phys. Gamma‐Ray Bursts 2018.

[57] R. E. Sari , T. Piran , R. Narayan , Astrophys. J. 1998, 497, L17.

[58] B. R. McNamara , P. E. J. Nulsen , Annu. Rev. Astron. Astrophys. 2007, 45, 117.

[59] R. A. Laing , A. H. Bridle , Mon. Not. R. Astron. Soc. 2002, 336, 328.

[60] M. C. Begelman , R. D. Blandford , M. J. Rees , Rev. Mod. Phys. 1984, 56, 255.

[61] B. Shen , Y. Li , M. Y. Yu , J. Cary , Phys. Rev. E 2007, 76, 055402. 10.1103/PhysRevE.76.055402 18233710

[62] Z. Gong , Y. Shou , Y. Tang , R. Hu , J. Yu , W. Ma , C. Lin , X. Yan , Phys. Rev. E 2020, 102, 013207. 32795002 10.1103/PhysRevE.102.013207

[63] Z. Gong , S. Cao , J. P. Palastro , M. R. Edwards , Phys. Rev. Lett. 2024, 133, 265002. 39879010 10.1103/PhysRevLett.133.265002

[64] X. Zheng , S. Zhu , X. Zhang , B. Shen , Opt. Express 2021, 29, 41121.

[65] K. M. Ferrière , Rev. Mod. Phys. 2001, 73, 1031.

[66] S. P. Reynolds , B. M. Gaensler , F. Bocchino , Space Sci. Rev. 2012, 166, 231.

[67] R. A. Daly , Astrophys. J. 2019, 886, 37.

[68] L. Ferrario , D. De Martino , B. T. Gänsicke , Space Sci. Rev. 2015, 191, 111.

[69] A. P. Igoshev , S. B. Popov , R. Hollerbach , Universe 2021, 7, 351.

[70] S. Fujioka , Z. Zhang , K. Ishihara , K. Shigemori , Y. Hironaka , T. Johzaki , A. Sunahara , N. Yamamoto , H. Nakashima , T. Watanabe , H. Shiraga , H. Nishimura , H. Azechi , Sci. Rep. 2013, 3, 7. 10.1038/srep01170 PMC3558719 23378905

[71] J. L. Peebles , J. R. Davies , D. H. Barnak , F. Garcia‐Rubio , P. V. Heuer , G. Brent , R. Spielman , R. Betti , Phys. Plasmas 2022, 29, 080501.

[72] M. Murakami , J. J. Honrubia , K. Weichman , A. V. Arefiev , S. V. Bulanov , Sci. Rep. 2020, 10, 16653. 33024183 10.1038/s41598-020-73581-4 PMC7538441

