# Supplementary material for: Proton Acceleration with Relativistic Electromagnetic Shock
Source: Adv Sci (Weinh). 2025 Jun 5;12(30):e03538. doi: 10.1002/advs.202503538 (PMC12376513; doi:10.1002/advs.202503538)
Supplement: Supplementary file 1 — Supporting Information [file ADVS-12-e03538-s001.docx]

Supporting Information

**Proton acceleration with relativistic electromagnetic shock**

Ting Xiao, Xiaomei Zhang, Fanqiu Kong, Xiaolong Zheng, Zheng Gong^†^ and Baifei Shen^*^

T. Xiao, X. Zhang, F. Kong, X. Zheng, B. Shen

Department of Physics

Shanghai Normal University

Shanghai 200234, China

*E-mail: bfshen@shnu.edu.cn

Z. Gong

CAS Key Laboratory of Theoretical Physics

Institute of Theoretical Physics

Chinese Academy of Sciences

Beijing 100190, China

^†^E-mail: zgong92@itp.ac.cn

1. The key to whether the mechanism is effective: the ratio of plasma kinetic energy density to magnetic energy density.

The plasma density is important to ensure the effectiveness of this mechanism. The plasma kinetic energy should be greater than magnetic energy to satisfy the criterion for realizing the magnetic field boosting. If the plasma density is too low, the magnetic field boosting cannot be realized. Assuming the plasma densities $n_{0}$ of ${10}^{16}\mathrm{cm}^{-3}$, ${10}^{18}\mathrm{cm}^{-3}$, ${10}^{20}\mathrm{cm}^{-3}$, ${10}^{22}\mathrm{cm}^{-3}$and ${10}^{23}\mathrm{cm}^{-3}$ with a velocity of $v=0.925c$, the corresponding plasma proton kinetic energy density densities are approximately ${2.4\times10}^{12} J/m^{3}$, ${2.4\times10}^{14} J/m^{3}$, ${2.4\times10}^{16} J/m^{3}$, ${2.4\times10}^{18} J/m^{3}$ and ${2.4\times10}^{19} J/m^{3}$, respectively. If only electrons are considered, the corresponding kinetic energy densities are approximately ${1.3\times10}^{9} J/m^{3}$, ${1.3\times10}^{11} J/m^{3}$, ${1.3\times10}^{13} J/m^{3}$, ${1.3\times10}^{15} J/m^{3}$ and ${1.3\times10}^{16} J/m^{3}$, respectively. As the strength of magnetic field is 40000 T, the magnetic energy density is $6.4\times{10}^{14} J/m^{3}$. The latter two density cases (${10}^{22}\mathrm{cm}^{-3}$, ${10}^{23}\mathrm{cm}^{-3}$) meet the condition that the kinetic energy is greater than the magnetic energy. We performed the 1D PIC simulation (see Figure S1) for the plasma density $n_{0}$ is ${10}^{22}\mathrm{cm}^{-3}$ and ${10}^{23}\mathrm{cm}^{-3}$, The plasma’s velocity is $0.925c$, which thickness is 240 nm starting from$x=10\lambda_{0}$. The coordinate in the co-moving frame of the plasma can be written as $\xi=x-v_{p}t$. A magnetic field with 40000 T along the *z*-axis in the region $x>10\lambda_{0}$. As shown in Figure S1 c, d, for plasma densities exceeding ${10}^{22} \mathrm{cm}^{-3}$, the magnetic field boosting can be obtained.


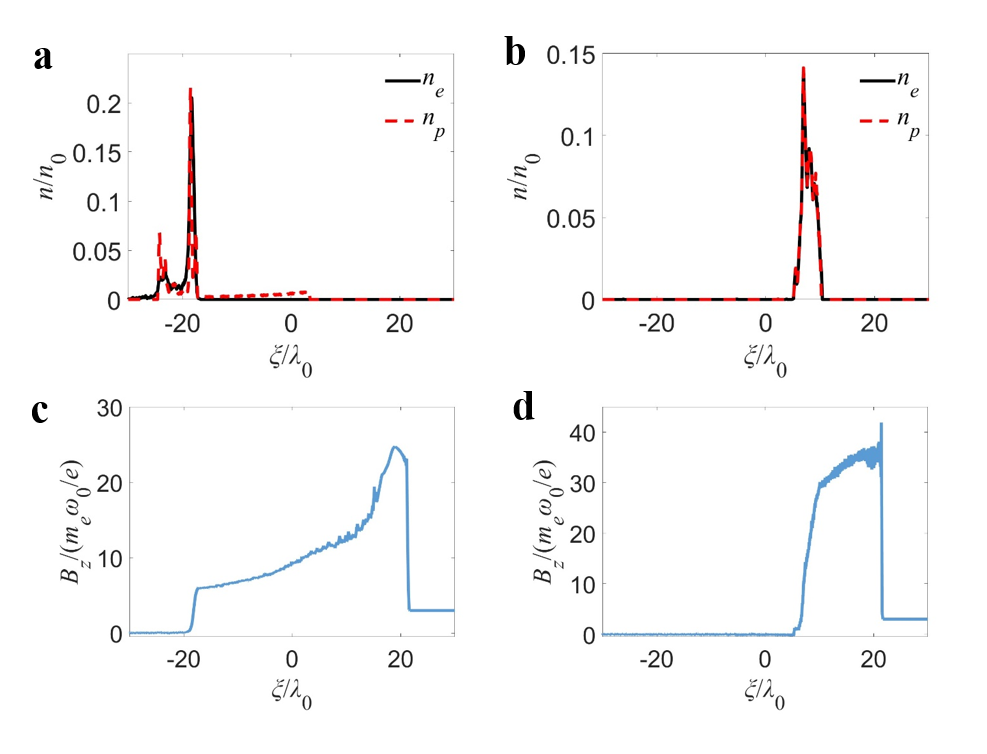


Figure S1 a) The density and c) magnetic field distribution with the initial plasma density $n_{0}={10}^{22}\mathrm{cm}^{-3}$ at $t=150T_{0}$. b) The density and d) magnetic field distribution with the initial plasma density $n_{0}={10}^{23}\mathrm{cm}^{-3}$ at $t=150T_{0}$. With the initial plasmas velocity $0.925c$, the plasma thickness is 240 nm starting from$x=10\lambda_{0}$. The coordinate in the co-moving frame of the plasma is written as $\xi=x-v_{p}t$. A magnetic field with 40000 T along the z-axis in the region $x>10\lambda_{0}$. The black line presents the electron density $n_{e}$, while the red dashed line shows the proton density $n_{p}$.

1. Preliminary 2D simulations indicate that the flying mirror can last for 1 fs and accelerate protons.

To check the robustness of our mechanism of multi-dimensional (2D) effects, we have performed 2D PIC simulations with the normalized amplitude of the laser (circular polarization) $a_{0}=150$, the focal spot radius is $40\lambda_{0}$ with fourth-order ultra-Gaussian intensity distribution, the trapezoidal laser time profile ($3T_{0}-10T_{0}-3T_{0}$), the plasma electron density $n_{e}=200n_{c}$, and the plasma thickness is 240 nm. The plasma is located at $10\lambda_{0}$, which transverse region is from $-45\lambda_{0}$ to $45\lambda_{0}$, The magnetic field is 40000 T, which is in the whole simulation region with $x>10\lambda_{0}$, $-50\lambda_{0}<y<50\lambda_{0}$. The momentum of the externally injected protons are $p_{x,in}=0.4$ and $p_{y,in}=0$, with their positions distributed in the range $250\lambda_{0}<x<350\lambda_{0}$. As shown in Figure S2, one can find that the laser-driven flying mirror can be maintained during an amazing timescale of one picosecond.


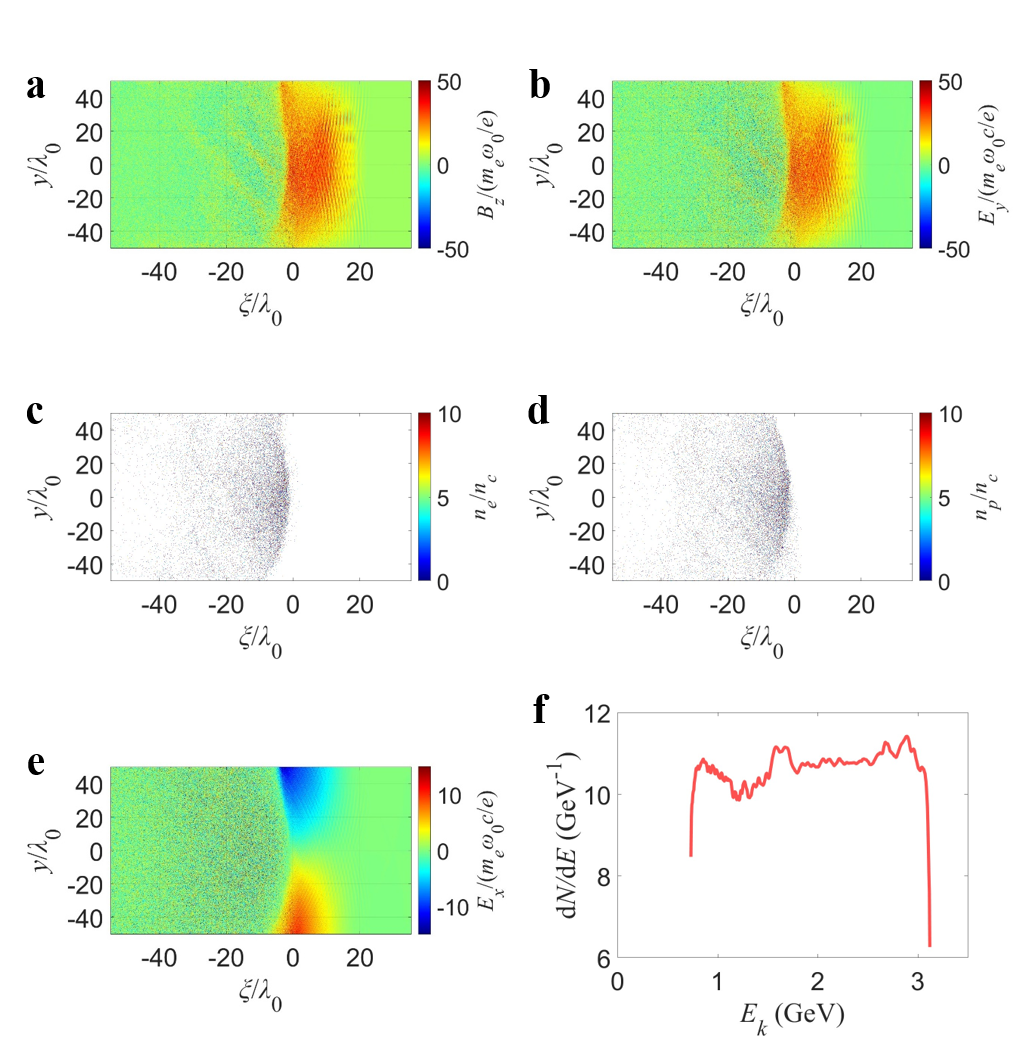


Figure S2 Two-dimensional PIC simulation results. a) the magnetic field $B_{z}$ (shown as color code) in the *z*-direction. b) the electric field $E_{y}$ (shown as color code) in the *y*-direction. c) and d) show the plasma density distribution (shown as color code) of electrons and protons, respectively. e) the electric field $E_{x}$ (shown as color code) in the *x*-direction at $t=350T_{0}$, when protons enter the electromagnetic shock wave. f) the energy spectral distribution of proton beam at the end of acceleration with $t=575T_{0}$. The coordinate in the co-moving frame of the plasma is written as $\xi=x-v_{p}t$.

The maximum proton energy obtained from two-dimensional PIC simulation is around 3 GeV [Figure S2 f], which is lower than the theoretically predicted proton energy of about 10 GeV for the same parameters. The discrepancy is because, on the one hand, the simulated electric field $E_{y}$ [Figure S2 b] resulting from the magnetic compression is weaker than that from the theoretical prediction due to the transverse inhomogeneity of the flying mirror (resulting from transverse instabilities). On the other hand, the non-uniform magnetic field $B_{z}$ in the *y* direction impacts the proton dynamics in the *x*-direction, which affects the acceleration direction of the protons. This gap (mainly caused by transverse instability) between simulation results and theoretically predicted proton energy is expected to be further narrowed by improved schemes, such as the use of ultra-Gaussian large focal spot lasers, special structure targets, and flying focus lasers.
